# Supplementary material for: Crowdfunding for complementary and alternative medicine: What are cancer patients seeking?
Source: PLoS One. 2020 Nov 20;15(11):e0242048. doi: 10.1371/journal.pone.0242048 (PMC7679016; doi:10.1371/journal.pone.0242048)
Supplement: S1 Appendix — (DOCX) [file pone.0242048.s001.docx]

S1 Appendix. CAM Search Terms.

| **CAM Treatment** | **Primary Search** | **Alternate-Related Searches** |
| --- | --- | --- |
| 714X | 714x AND cancer |  |
| Acai | acai AND cancer |  |
| Acupuncture | acupunct* AND cancer |  |
| Alpha-linolenic Acid | alpha lino* AND cancer |  |
| Alpha-Tocopherol | alpha toco* AND cancer |  |
| Amygdalin | amygdalin AND cancer |  |
| Antineoplastions | Antineoplast* AND cancer |  |
| Antioxidants | antioxidant* AND cancer |  |
| Aromatherapy | aromatherap* AND cancer |  |
| Art therapy | "art therapy" AND cancer |  |
| Astralagus | astralagus AND cancer |  |
| Ayurveda | ayurveda AND cancer | ayurved* AND cancer |
| Beta Glucans | beta glucan* AND cancer |  |
| Biofeedback | biofeedback AND cancer |  |
| Bitter Orange | "bitter orange" AND cancer |  |
| Black Cohosh | black cohosh AND cancer |  |
| Bovine Cartilage | "bovine cartilage" AND cancer |  |
| Calcium | calcium AND cancer |  |
| Cancell | cancell AND cancer |  |
| Cannabis | cannabis AND cancer | marijuana AND cancer; “cbd” AND cancer; “cannabidiol” AND cancer |
| Cantron | cantron AND cancer |  |
| Cat’s Claw | "cat's claw" AND cancer |  |
| Chiropractic | chiropract* AND cancer |  |
| Coenzyme Q10 | coenzyme Q10 AND cancer |  |
| Coffee Enema | "coffee enema" AND cancer | "coffee enemas" AND cancer |
| Cognitive-behavioral therapy | "cognitive behavioral" AND cancer | cbt AND cancer; “cognitive behavioural” AND cancer |
| Colloidal Silver Products | colloidal AND cancer |  |
| Cranial Stimulation | "cranial stimulation" AND cancer |  |
| Cruciferous vegetables | cruciferous AND cancer |  |
| Dietary Supplements | "dietary supplements" AND cancer | "dietary supplement" AND cancer |
| Electroacupuncture | electroacupunct* AND cancer |  |
| Essential Oils | "essential oils" AND cancer | "essential oil" AND cancer |
| Essiac | essiac AND cancer |  |
| Fish Oil | "fish oil" AND cancer |  |
| Flor-Essence | "flor essence" AND cancer | “flor-essence” AND cancer |
| Gerson therapy | gerson* AND cancer |  |
| Ginseng | ginseng AND cancer |  |
| Goldenseal | goldenseal AND cancer |  |
| Green Tea | "green tea" AND cancer |  |
| Herbs and herbal extracts | herb* AND cancer |  |
| Homeopathy | homeopath* AND cancer |  |
| Hydrazine sulfate | hydrazine AND cancer |  |
| Hyperthermia | hyperthermia AND cancer |  |
| Hypnosis | hypno* AND cancer |  |
| Imagery | imagery AND cancer |  |
| Immunoaugmentative therapy | immunoaugment* AND cancer |  |
| Intercessory prayer | "intercessory prayer" AND cancer |  |
| Kava | kava AND cancer |  |
| Kelley/Gonzalez regimen/protocol | "kelley regimen" AND cancer | "kelley protcol" AND cancer; gonzalez regimen" AND cancer; "gonzalez protocol" AND cancer |
| Laetrile | laetrile AND cancer |  |
| Low dose naltrexone | naltrexone AND cancer |  |
| Lycopene | lycopene AND cancer |  |
| Macrobiotic diet | macrobio* AND cancer |  |
| Magnet therapy | "magnet therapy" AND cancer |  |
| Medicinal Mushrooms | medicinal AND mushroom* AND cancer |  |
| Meditation | meditation AND cancer |  |
| Melatonin | melatonin AND cancer |  |
| Milk Thistle | "milk thistle" AND cancer |  |
| Mind-Body Medicine | "mind body" AND cancer |  |
| Mistletoe | mistletoe AND cancer |  |
| Mixtures of tea polyphenols | "tea polyphenols" AND cancer |  |
| Modified Citrus Pectin | "citrus pectin" AND cancer |  |
| Multivitamin | multivitamin AND cancer |  |
| Music therapy | "music therapy" AND cancer |  |
| Naturopathy | naturopath* AND cancer |  |
| Newcastle Disease Virus | "newcastle disease" AND cancer |  |
| Noni | noni AND cancer |  |
| Omega-3 Fatty Acids | "omega 3" AND cancer |  |
| Osteopathy | osteopath* AND cancer |  |
| PC-SPES | "PC SPES" AND cancer |  |
| Primrose Oil | primrose AND cancer |  |
| Probiotics | probiotic* AND cancer |  |
| Protocel | protocel* AND cancer |  |
| Pulsed electromagnetic fields | electromagnet* AND cancer |  |
| Qi gong | "qi gong" AND cancer | qigong AND cancer |
| Red Algae | "red algae" AND cancer |  |
| Red Clover | "red clover" AND cancer |  |
| Red Sage | "red sage" AND cancer |  |
| Reflexology | reflexolog* AND cancer |  |
| Reiki | reiki AND cancer |  |
| Relaxation therapy | "relaxation therapy" AND cancer |  |
| SAMe (S-Adenosyl-L-Methionine) | adenosyl AND cancer | methionine AND cancer |
| Selenium | selenium AND cancer |  |
| Shark cartilage | "shark cartilage" AND cancer |  |
| Soy phytoestrogens | Soy AND phyto* AND cancer |  |
| Spiritual healing | "spiritual healing" AND cancer |  |
| Spirituality | spirituality AND cancer |  |
| Sun’s Soup | "sun's soup" AND cancer | "suns soup" AND cancer |
| T'ai chi | "tai chi" AND cancer | "t'ai chi" AND cancer |
| Tea | tea AND cancer |  |
| Therapeutic massage | "therapeutic massage*" AND cancer |  |
| Therapeutic touch | "therapeutic touch*" AND cancer |  |
| Thunder God Vine | thunder god vine AND cancer |  |
| Tibetan Medicine | "tibetan medicine" AND cancer |  |
| Traditional Chinese Medicine (TCM) | "chinese medicine" AND cancer | TCM and Cancer |
| Tripterygium wilfordii | tripterygium wilfordi* AND cancer |  |
| Turmeric | turmeric AND cancer |  |
| Vegetarianism | vegetarian* AND cancer |  |
| Vitamin A | vitamin* AND cancer |  |
| Vitamin B | vitamin* AND cancer |  |
| Vitamin C | vitamin* AND cancer |  |
| Vitamin D | vitamin* AND cancer |  |
| Vitamin E | vitamin* AND cancer |  |
| Vitamin K | vitamin* AND cancer |  |
| Vitamin Supplements | vitamin* AND cancer |  |
| Vitamins | vitamin* AND cancer |  |
| Yoga | yoga AND cancer |  |
| Yoga asanas | yoga asan* AND cancer |  |
| Zyflamend | zyflamend AND cancer |  |
